# Use of Split-Thickness Skin Grafts and Autologous Skin Cell Suspension in a Case of Extensive Fournier Gangrene

**DOI:** 10.7759/cureus.62639

**Published:** 2024-06-18

**Authors:** Connor J English, Oluwafolaranmi E Sodade, Cindy L Austin, Krisi A Causa

**Affiliations:** 1 Trauma Surgery, A.T. Still University Kirksville College of Osteopathic Medicine, Kirksville, USA; 2 Trauma Research, Mercy Hospital Springfield, Springfield, USA; 3 Trauma & Burn Surgery, Mercy Hospital Springfield, Springfield, USA

**Keywords:** autologous skin cell suspension, split thickness skin graft, marsupialization, necrotizing soft tissue infection (nsti), fournier gangrene

## Abstract

Fournier gangrene (FG) is a life-threatening necrotizing soft-tissue infection of the perineum and external genitalia, which primarily occurs in obese, diabetic males. The mainstay of treatment is source control via early aggressive surgical excision. Wide surgical excision can result in significant soft tissue defects that can be disfiguring and difficult to close. The most common method of closure is split-thickness skin grafting (STSG). Recently, autologous skin cell suspension (ASCS) technology has been used in addition to STSG to provide better wound healing and closure. This patient experienced excellent wound progression, following FG, through the application of ASCS with STSG, despite challenges related to the wounds, anatomical location, comorbidities, size, and the patient’s medical history.

## Introduction

Fournier gangrene (FG) is a life-threatening necrotizing soft-tissue infection (NSTI) of the perineum and external genitalia [1]. Though all NSTI are rare, with an estimated incidence of 8.7-10.3 per 100,000 persons [2], FG is one of the most common types of NSTI, with an incidence 1.6 per 100,000 males, occurrence in females is exceedingly rare [3]. The reported mortality rate of FG varies widely; however, approximately 20% is recognized as an acceptable measure [4].

The most common risk factors for all NSTI include diabetes mellitus, obesity, chronic kidney failure, alcoholism, and injection drug use [3,5,6]. FG falls under the umbrella of Type I NSTI, which are polymicrobial in nature and often begin in the setting of chronic wounds or skin defects [6]. Bacterial enzymes and endotoxins in the subcutaneous tissues cause local tissue destruction and obliterative microvascular endarteritis, propagating further ischemia and necrosis [7]. Upon initial presentation, the superficial appearance of the infected area is rarely representative of the magnitude of hypodermic destruction. This can lead to confusion with more benign disease processes such as impetigo, cellulitis, or other superficial soft-tissue infections [8]. When signs of systemic infection present with questionable skin or soft tissue findings, a high degree of clinical suspicion is needed to make the diagnosis of NSTI. “Pain out of proportion to the physical exam” is a classic symptom. FG, specifically, tends to present with scrotal swelling, drainage, crepitus, or skin breakdown [8,9]. Timely diagnosis is imperative to preserve tissue and prevent decompensation to septic shock, disseminated intravascular coagulation, or death [3,5,9].

The mainstay of treatment is source control via early aggressive surgical excision alongside broad-spectrum antibiotics [6,9,10]. Once infection control has been achieved, more so in FG than other NSTI, definitive coverage of the tissue is complicated by body habitus, moisture and friction of intertriginous areas, and wound contamination [10]. Temporary diverting colostomy is often necessary to prevent fecal contamination of the dehiscent wound. Numerous coverage and wound care techniques have been described in the literature. Primary closure, healing by secondary intention, split- or full-thickness skin grafts, and local advancement flaps are some of the most common methods of closure, all of which are dependent on wound size [6,10,11]. Additional procedures are often required for coverage of the testicles such as scrotal flaps or testicular transposition [11,12]. The best option for each patient is dependent on wound dimensions, body mass index (BMI), comorbid conditions, and the skill and preference of the surgeon [10]. We present a case of extensive FG in a morbidly obese male with exceptional post-excision wound recuperation through the combined use of split thickness skin grafting (STSG) and autologous skin cell suspension (ASCS). 

## Case presentation

**Figure 1:**
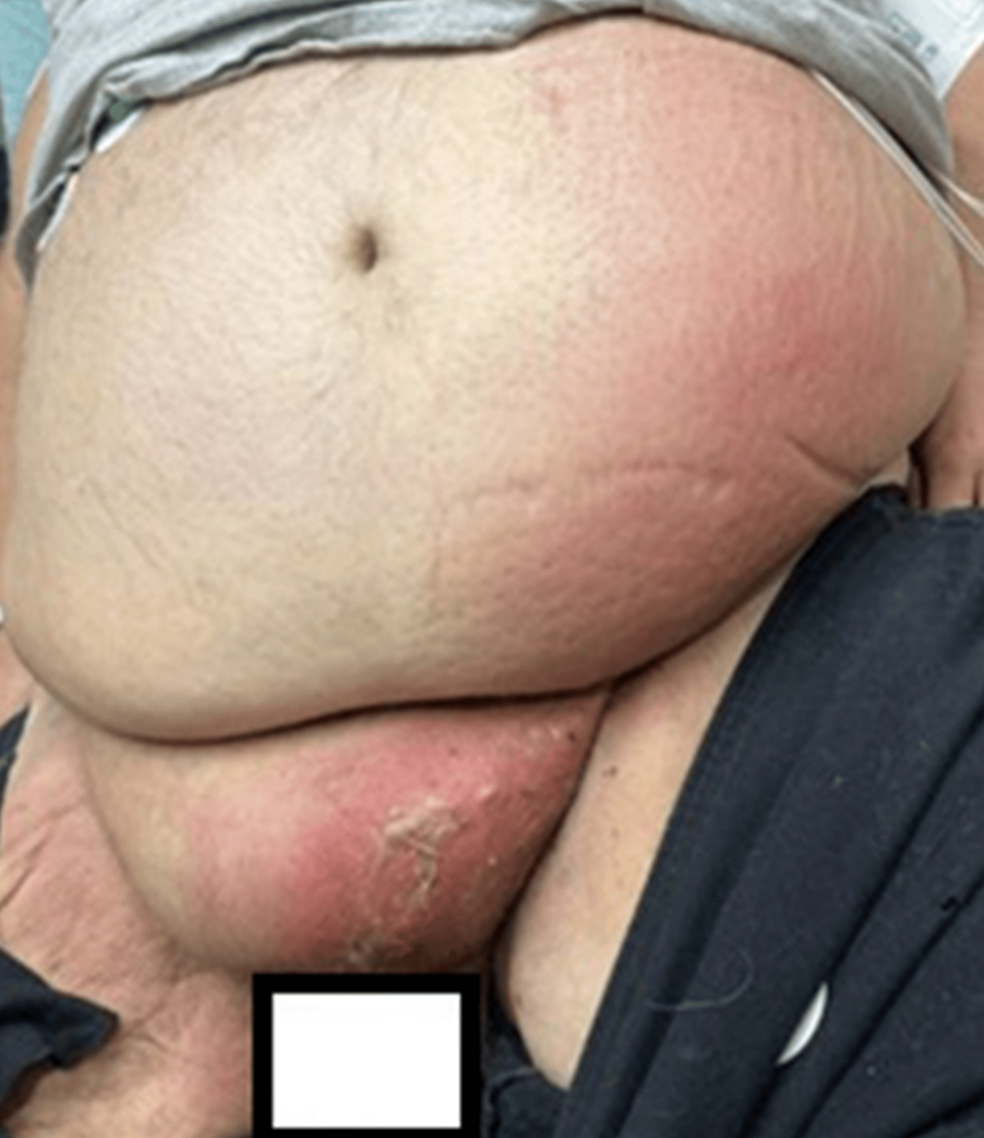
Abdominal and scrotal appearance on Emergency Department presentation

A 37-year-old male with a medical history of morbid obesity (BMI 52), type 2 diabetes mellitus, tobacco use, and necrotizing fasciitis requiring numerous surgical debridements presented to the emergency department (ED) with a complaint of left scrotal pain and swelling for one week, with new onset redness of his lower abdomen for one day. He reported episodes of incontinence and dysuria, but without fever, chills, nausea, or vomiting. Physical exam revealed significant scrotal swelling, erythema, and tenderness which extended to the left lower quadrant (LLQ) of the abdomen. Induration and desquamation of the scrotal and suprapubic skin were present (Figure 1). The inferior and posterior portions of the scrotum appeared black and necrotic. The patient met criteria for severe sepsis based on tachycardia, tachypnea, and likely infection source; he was not in shock in the ED. 

**Figure 2:**
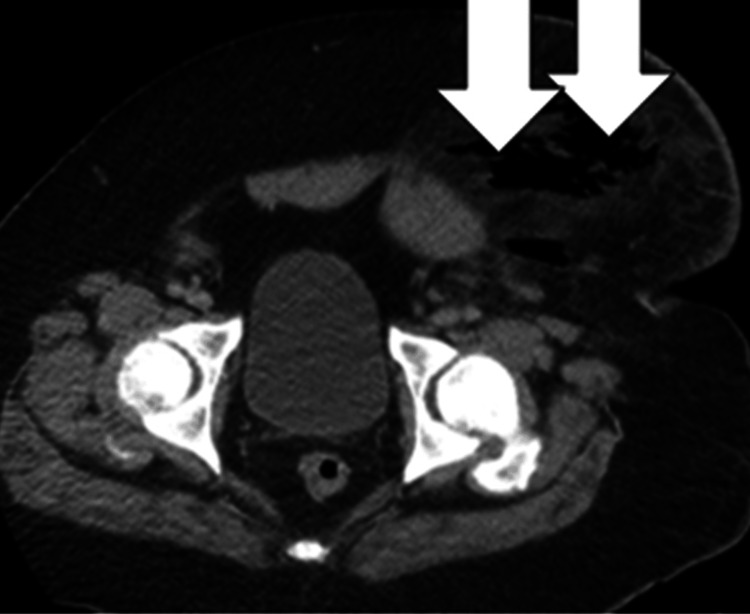
Evidence of subcutaneous gas in the left abdominal wall on CT scan in the Emergency Department

Blood cultures were collected and intravenous (IV) vancomycin, clindamycin, and cefepime were initiated. Computed tomography (CT) of the abdomen and pelvis revealed significant subcutaneous gas in the scrotum and left lower abdominal wall (Figure 2). Acute Care Surgery was consulted for the diagnosis of FG with severe sepsis. Following the surgeon’s evaluation, the patient was taken emergently to the operating room (OR) for exploration and debridement.

**Figure 3:**
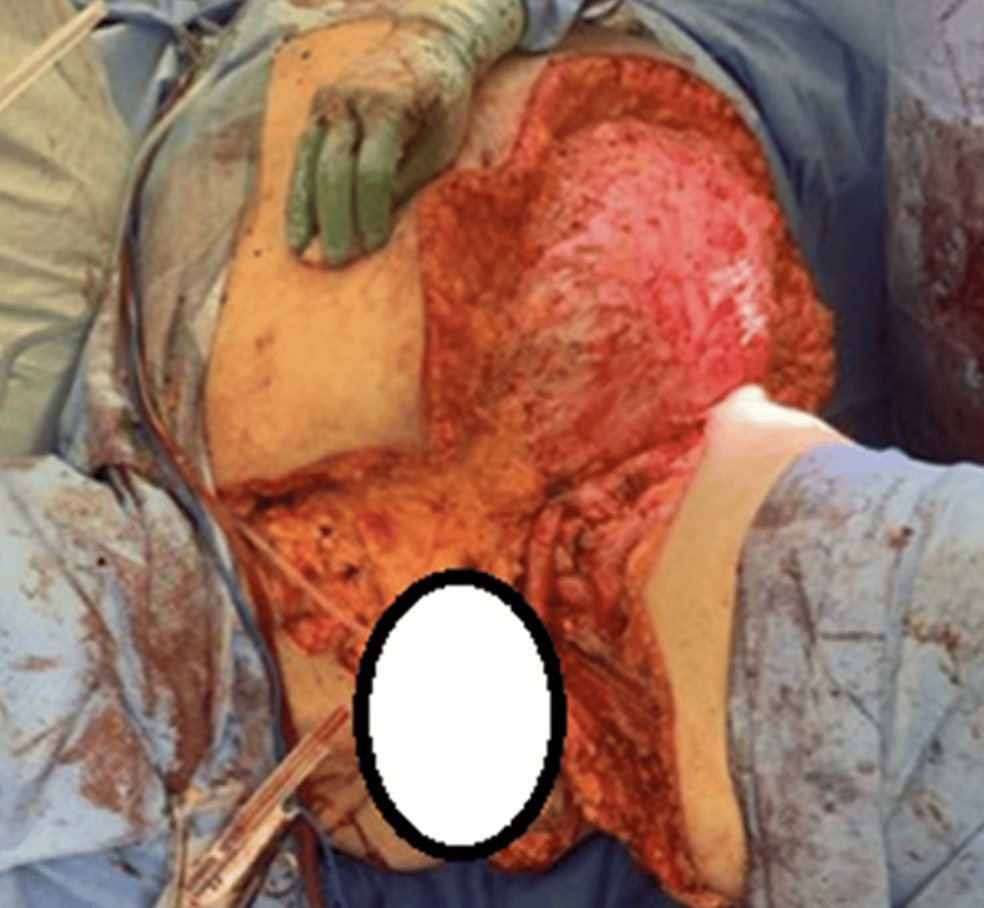
Post excision, hospital day one.

**Figure 4:**
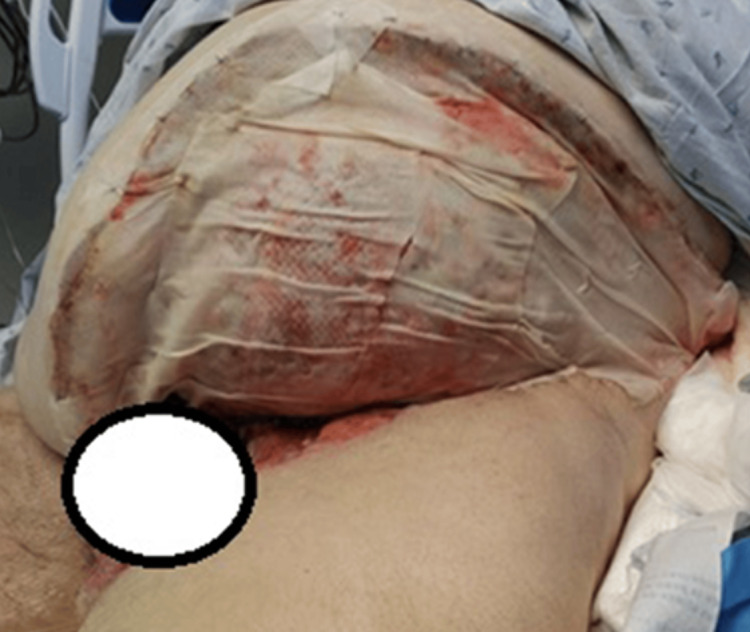
Fish-skin substitute over debrided abdominal wound, hospital day four.

During the initial operation on hospital day one, nonviable tissue was removed from the left thigh, perineum, scrotum (removed in its entirety), suprapubic region, and abdominal wall. A total of 4,620 square centimeters of skin, subcutaneous tissue, and fascia were removed (Figure 3). Urology was consulted for a potentially threatened left testis, otherwise all remaining tissue appeared viable after the procedure. Upon transfer to the trauma intensive care unit (ICU), the patient experienced glycemic control issues which required aggressive IV insulin management and septic shock requiring pressor support. On hospital day three, the patient returned to the OR for further debridement and removal of 5,060 square centimeters of tissue from the left abdominal wall and thigh, right groin, suprapubic region, peritoneum, and penis. A 2,520 square centimeter piece of fish skin substitute was applied to the large abdominal wound to promote granulation and protect the wound bed (Figure 4). Blood flow was not highlighted upon repeat Doppler evaluation of the vasculature of the left testis and left orchiectomy was performed as well. The patient was stable enough for transfer from the ICU to the burn and wound unit/center on hospital day four.

The patient had no major adverse events or new complaints on hospital days five through 16. He underwent routine dressing changes and began inpatient physical and occupational therapies. On hospital day 17, the patient returned to the operating room for further debridement of 1,200 square centimeters of the abdominal wall, removal of portions of non-adherent skin substitute, marsupialization of the right testis, and STSG of the penis using an autograft harvested from left thigh. The patient experienced minimal issues with the penis graft. On hospital day 23, a wound vacuum assisted closure (VAC) device was placed in the left groin and perineal area to promote further granulation and wound irrigation. The patient continued physical and occupational therapies and regular wound VAC and dressing changes in the hospital setting with no major complications or setbacks.

**Figure 5:**
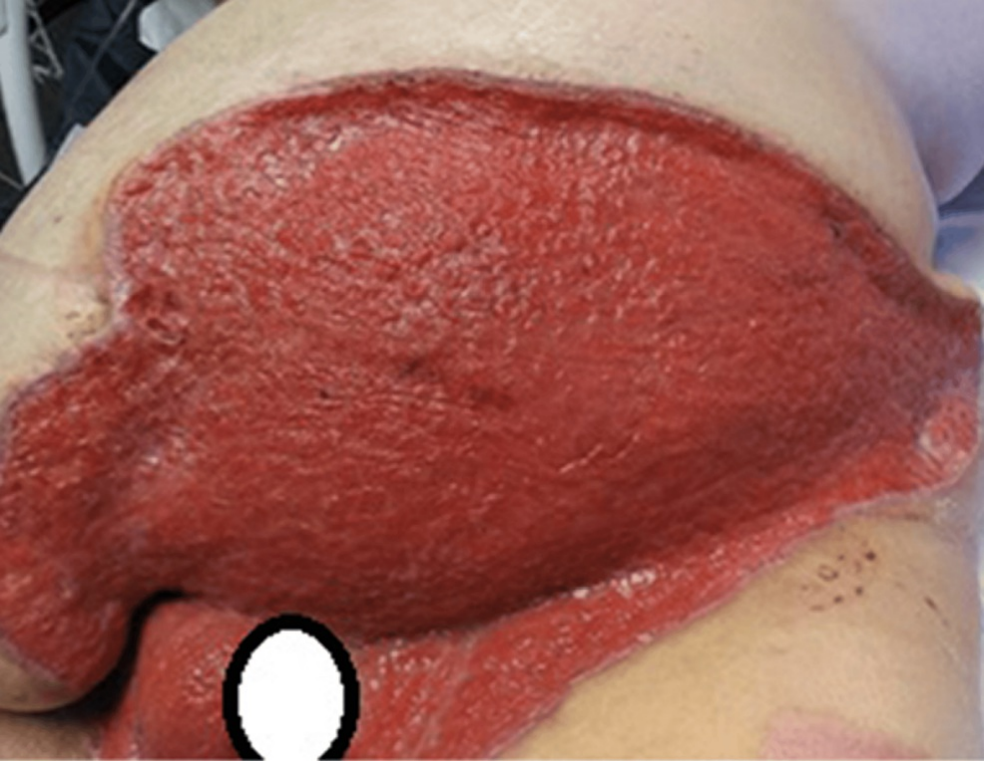
Granulated wound bed prior to grafting, hospital day 43.

**Figure 6:**
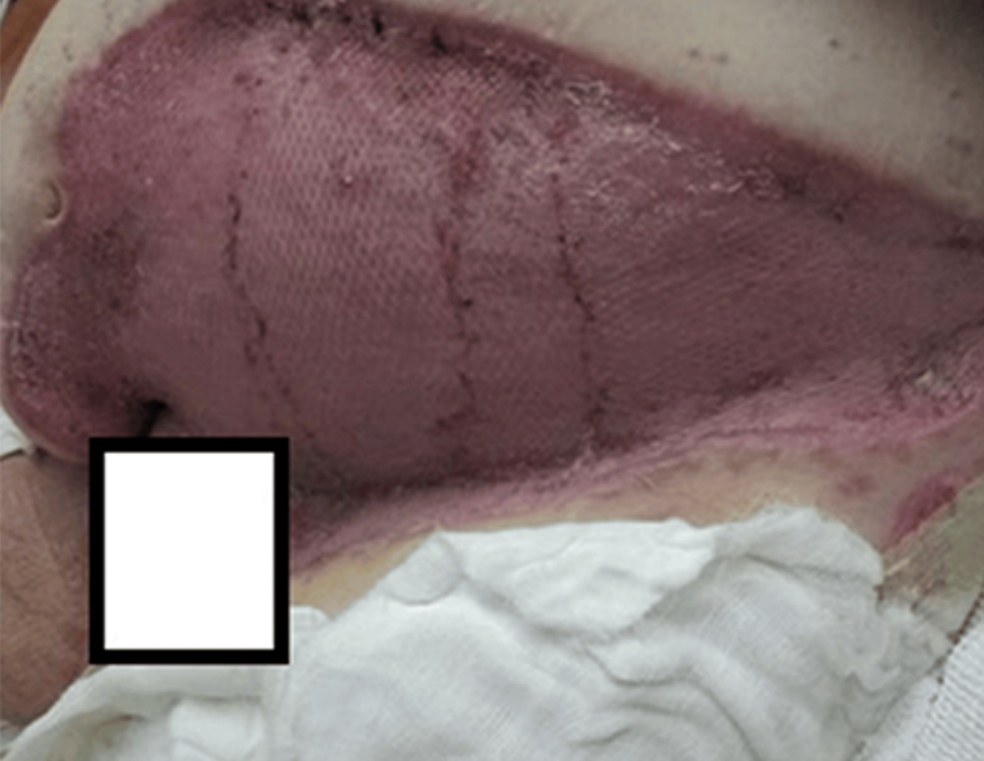
Grafted wound on hospital day 49, approximately one week following grafting

**Figure 7:**
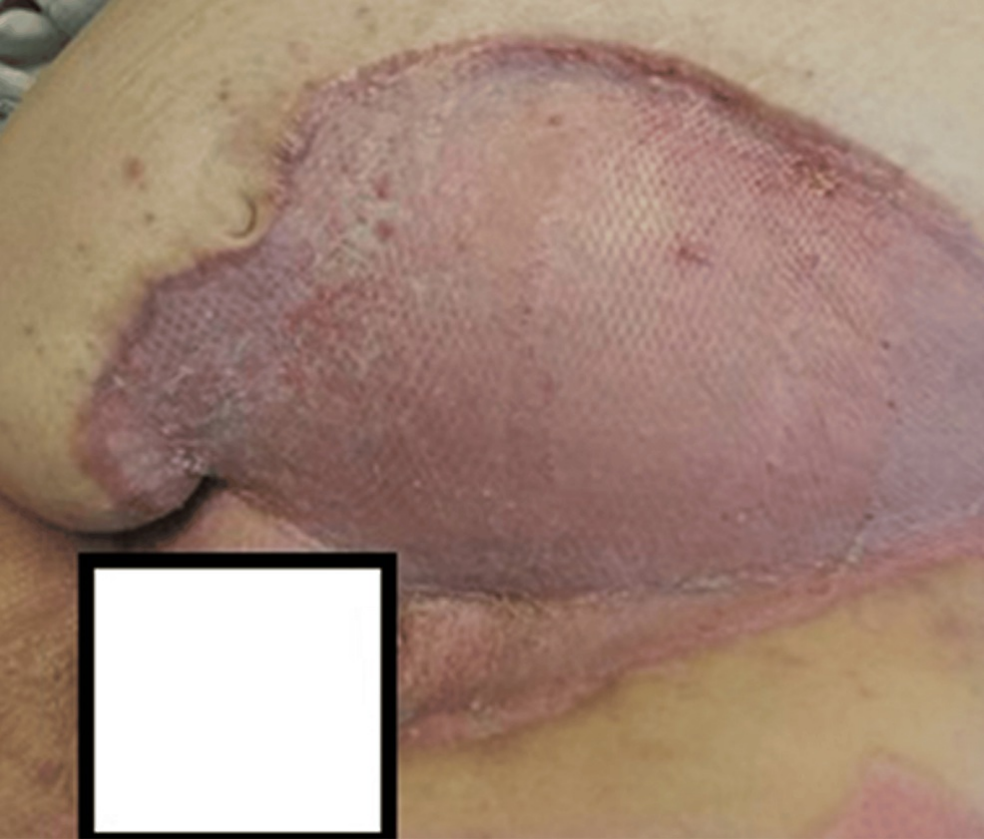
Grafted wound on hospital day 57, approximately two weeks following grafting

On hospital day 43, the patient returned to the OR for a definitive grafting procedure (Figure 5). All wounds were debrided in an excisional fashion using water jet technology until a healthy, bleeding wound bed was obtained. Approximately 4,000 square centimeters of epidermal split-thickness skin grafts were harvested from the bilateral thighs and right abdominal wall using a dermatome set at 0.009 inches with a four-inch guard. All the grafts were meshed in a 2:1 ratio and secured over the abdominal, suprapubic, thigh, and perineal wounds using staples. To reduce the risk of graft failure, particularly in intertriginous areas, portions of autograft were used to create an ASCS that was then sprayed over all of the grafts and donor sites. All areas were then dressed with a layer of clear wound dressing and a layer of two-sided contact dressing. Full dressing takedown on hospital day 49 revealed excellent wound progress and no obvious areas of graft failure (Figure 6). Other than some intermittent issues with urinary hesitancy which did not require intervention, the patient progressed nicely with therapies and had no new concerns days 50 through 57. The patient was discharged home on hospital day 57 after he refused further inpatient rehabilitation. The patient was encouraged to maintain good glycemic control and eliminate nicotine-containing products to allow for optimal healing (Figure 7).

**Figure 8:**
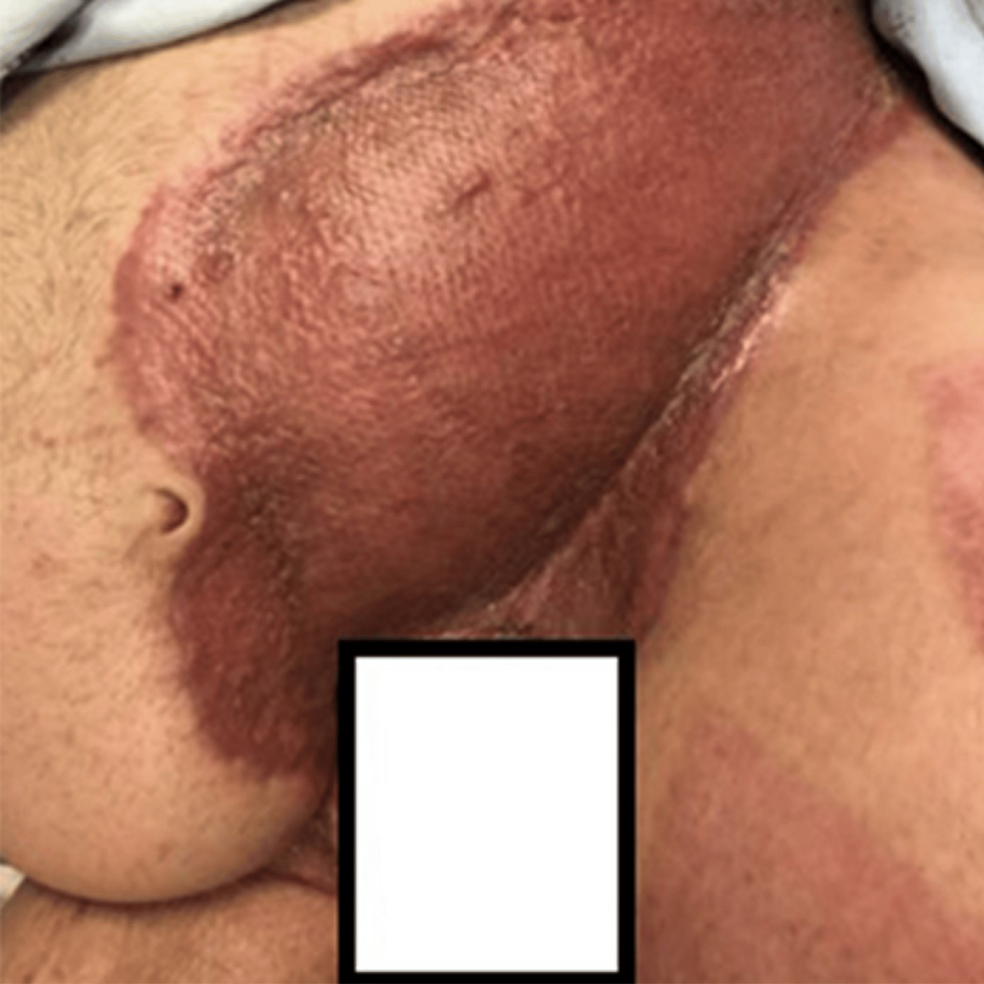
Four-week post-discharge follow-up, approximately six weeks since grafting.

The patient followed-up in the wound clinic two weeks after discharge with no major complaints and continued improvement of his grafts. He returned four weeks after discharge with a complaint of left groin and hip tightness, but no graft issues. The patient was given mobility exercise education and continued nicotine cessation encouragement. Final follow-up eight weeks after discharge revealed some dryness and friction-related blistering in the perineum for which a moisturizing plan and improved hygiene were discussed (Figure 8). Overall, graft coverage and healing was successful despite challenges associated with the patient’s body habitus and extent of his wounds. In this patient, application of the autologous skin cell suspension in conjunction with STSG seemed to improve the success and rapidity of graft take and overall wound healing.

## Discussion

FG is a rapidly progressing NSTI with a high mortality rate [4]. Aggressive surgical excision with broad-spectrum antimicrobial therapy is the only appropriate treatment strategy [6,10]. Excision of necrotic tissue is considered sufficient when blunt dissection can no longer easily separate subcutaneous tissue from the underlying fascia. Most patients require numerous debridement procedures to ensure that progression of necrosis is halted and additional compromised tissue is swiftly removed [12]. The repeat operations result in large open wounds which require complex soft tissue and skin coverage [6,10]. Most surgeons opt for utilization of STSG to cover the granulated wound bed, as the massive loss of soft tissue allows few other options. Healing, granulation, and definitive coverage of the wound bed in a FG case is complicated by location and body habitus [13]. Several therapies are used in addition to STSG to promote the healing process. Wound VAC devices applied over the top of the grafted wound are thought to promote healing through the use of negative (subatmospheric) pressure. Hyperbaric oxygen therapy (HBOT) is used to encourage healing and reduce infection risk by hyper-oxygenating the tissue [13,14].

Literature discussing the use of ASCS or similar products in the treatment of FG tissue defects is minimal, though its efficacy as an adjunctive therapy with STSG has been noted. ASCS used in addition to meshed-STSG versus STSG alone has shown an increased percentage of wound closure at eight weeks, and a decreased amount of donor skin required to cover wounds of a similar size [15]. The suspension is prepared point-of-care from a small piece of autograft that is mixed into a solution; this creates a spray that can be applied over the top of the grafted areas and donor sites. One square centimeter of the patient's skin can create enough suspension to cover eighty square centimeters of tissue. The ASCS promotes rapid re-epithelialization, increasing the likelihood of graft-take and promoting overall healing of the wound bed [15]. ASCS also has the added benefit of acting like an adhesive, which has been utilized for some time [14] to maintain STSG contact with the wound surface [15].

 In this case, the care team determined that adding ASCS to the meshed STSG would provide the best chance for wound closure and minimize the risk of massive graft failure which was a concern due to the location of the grafts, particularly in the groin and perineal areas. Given the patient’s relevant history of diabetes mellitus and tobacco use, ASCS offered additional epithelial regenerative support that may be compromised given his comorbidities. The patient experienced excellent progression upon the first complete evaluation of his wounds just six days after the complete grafting procedure. He showed no evidence of graft failure or other wound related complications by day 14 and was discharged home with wound care instructions. The patient experienced some perineal wound breakdown at the eight-week follow-up appointment which seemed to be related to cleanliness and the type of clothing he was wearing. He was educated on more specific wound care instructions. The patient also showed some evidence of a right groin contracture band that may need to be addressed later. It was not yet hindering his function, though it was causing some discomfort with specific hip movements. Overall, the use of ASCS with STSG in this patient seemed to promote better wound and graft healing despite challenges with wound location and size, and patient comorbidities.

## Conclusions

Traditionally, treatment options for each specific patient are determined by factors such as wound dimensions, BMI, comorbidities, skills, and preferences of the surgeons. In this case, the holistic surgical approach and consults across multi-specialties contributed significantly towards an improved clinical outcome.

Based on this, the collaborative efforts of the clinical teams and the use of ASCS as a complementary treatment to STSG may have provided additional wound healing support in patients with challenging anatomy, complex wounds, and medical comorbidities. However, a long-term patient follow-up assessment is needed to evaluate possible adverse effects such as increased contracture risk.
